# Batteries Not Included: A Self-Powered Cardiac Pacemaker

**DOI:** 10.20900/mo.20190016

**Published:** 2019-08-20

**Authors:** Jae Hyung Cho, Eugenio Cingolani

**Affiliations:** Smidt Heart Institute, Cedars-Sinai Medical Center, Los Angeles, CA 90048, USA

Since the first electronic pacemaker was implanted in human in 1958, electronic pacemakers have undergone continuous refinement including miniaturization of the devices all the way to a standalone leadless pacemaker capable of right ventricular pacing [1]. Other pacing modalities such as cardiac resynchronization therapy and His-bundle pacing also are available for treatment of selected patients. Biological pacemakers have and continue to be tested in pre-clinical models as a “hardware free” alternative to electronic devices [2,3]. Despite advances in device technologies, there are still limitations of devices such as: infectious complications, over or under sensing/pacing, lack of true autonomic response, and need for generator replacements. One of the recent advances in device technology is the development of battery-less electronic devices that harvest energy from heart beats, muscle stretching, glucose oxidation and endocochlear potentials. In this Nature Communications article [4], Ouyang *et al*. demonstrated that a symbiotic cardiac pacemaker (powered by a triboelectric nanogenerator which harvest energy from cardiac motion) can successfully pace the heart in a porcine model of sinus arrest. This article not only tested the feasibility of a “self-powered” cardiac pacemaker but also brings hope for the future of next-generation pacemakers, which could potentially co-exist with the patients. The major benefits of this new technology are that we can potentially reduce the size of current generators and there is no need to replace the generator at the end of battery life.

By using a nanogenerator, which can harvest the native energy generated by cardiac motion, Ouyang *et al*. tested the feasibility of durable symbiotic pacemaker in a porcine model of sinus node dysfunction. The symbiotic cardiac pacemaker consists of three units: energy harvest unit, power management unit, and pacemaker unit. The triboelectric nanogenerator has many advantages: flexibility, biocompatibility, implantability and light weight. Superior energy output, biocompatibility and stability are the vital characteristic of the energy harvester. The energy harvested by the unit from cardiac motion was stored in a capacitor to drive the pacemaker. In the present work, the authors used adult male Yorkshire porcines and created transient sinus node dysfunction by hypothermia. The implanted symbiotic pacemaker succeeded in correcting sinus bradycardia by pacing the animals when needed. The premise behind the successful experiments of the authors was that the energy harvested from each cardiac motion (0.495 μJ) was greater than that required for endocardial pacing threshold (0.377 μJ). This interesting technology has the potential to apply to many medical devices (other than cardiac pacemakers) such as implantable cardiac defibrillators, deep brain stimulators, etc. This new technique provides a promising method to harvest, store, and discharge energy to pace the heart with the advantages of a wide choice of materials, high output, flexibility, and decreased size of the generator.

A couple of important issues should merit further clarification. First of all, although this technique is promising, it has limited applicability to pacemaker-dependent patients. If the patient is 100% pacemaker dependent, since the generator can only save a portion of the energy generated (limited by the capacitor), the pacemaker will not be able to store enough energy to pace the patient for the rest of his/her life. Also, pacemakers need additional energy to analyze the underlying rhythm, check sensitivity and change pacing thresholds as well as sending data to the remote monitoring systems. All of these functions will increase energy consumption and it is unclear how much more energy will be required to keep up with functionality of current pacemakers. Second, because of the concern that the nanogenerator does not harvest enough energy to pace, the pacemaker may need a back-up battery to store energy. This can increase the size of the generator, which can potentially increase complications and limit the benefit of this technology. Third, this new technique still does not protect electronic pacemakers from infectious complications and lack of autonomic nervous system response, issues that could only be solved if a biological pacemaker (currently in pre-clinical stages) ever advances to the clinic. Lastly, the main benefit of this technology is limited to avoiding multiple generator changes. As battery technologies continue to advance, battery life will likely extend and most patients may not require frequent generator changes. The concept of self-powered pacemaker is novel but with the current prototypes the advantages are marginal.

Nevertheless, the concept of a self-powered pacemaker represents an important advance in device technology, and the electrophysiology community (and our patients) look forward to using our own energy to keep the pace.
